# Temperature and WNK-SPAK/OSR1 Kinases Dynamically Regulate Antiviral Human GFP-MxA Biomolecular Condensates in Oral Cancer Cells

**DOI:** 10.3390/cells14130947

**Published:** 2025-06-20

**Authors:** Pravin B. Sehgal, Huijuan Yuan, Susan V. DiSenso-Browne

**Affiliations:** 1 Department of Cell Biology and Anatomy, New York Medical College, Valhalla, NY 10595, USA; 2 Department of Medicine, New York Medical College, Valhalla, NY 10595, USA; 3 Touro College of Dental Medicine at New York Medical College, Hawthorne, NY 10532, USA

**Keywords:** pathogenesis of oral cancer, anatomical sites of oral cancer, hypotonic and temperature stresses, structure of biomolecular condensates, human myxovirus resistance protein (MxA/Mx1), WNK-SPAK/OSR1 kinases, uncrowding/recrowding

## Abstract

Phase-separated membraneless biomolecular condensates in the cytoplasm and nucleus are now recognized to play a major role in modulating diverse functions in mammalian cells, and contribute to cancer pathogenesis through dysregulated function of condensates of transcription factors such as STAT3 and fusion oncoproteins. Oral cancer, the sixth most prevalent malignancy worldwide, in the absence of overt causes such as tobacco or alcohol, most frequently occurs in a U-shaped zone (floor of mouth, side of tongue, anterior fauces and retromolar region) reflecting the path of liquid transit through the mouth. The cellular basis for this “high-risk” zone and the biochemical mechanisms used by oral cells to combat repetitive tonicity and temperature stresses are incompletely understood. We had previously observed that at 37 °C, in OECM1 oral carcinoma cells, cytoplasmic condensates of antiviral human GFP-MxA GTPase disassembled within 1–2 min of exposure of cells to saliva-like one-third hypotonicity, and underwent “spontaneous” reassembly in the next 5–7 min. Moreover, hypotonic beverages (water, tea, coffee), investigated at 37 °C, triggered this condensate cycling. In the present studies we investigated whether this process was temperature sensitive, representative of cold vs. warm drinks. We observed a slowing of this cycle at 5 °C, and speeding up at 50 °C. The involvement in this disassembly/reassembly process of WNK-SPAK/OSR1 serine-threonine kinase pathway, best studied for regulation of water and Na, K and Cl influx and efflux in kidney tubule cells, was evaluated by us in *oral* cells using pathway inhibitors WNK463, WNK-IN-11 and closantel. The pan-WNK inhibitor WNK463 inhibited hypotonicity-driven condensate disassembly, while the SPAK/OSR1 inhibitor closantel markedly slowed reassembly. Unexpectedly, the WNK1-selective inhibitor (WNK-IN-11), triggered a dramatic and rapid (within 1 h) spheroid to fibril transition of GFP-MxA condensates in live cells, but without affecting MxA antiviral function. The new data suggest a novel hypothesis for the anatomic localization of oral cancer in the U-shaped “high-risk” zone in the mouth: dysfunction of biomolecular condensates in oral cells along the beverage transit pathway through the mouth due to repetitive tonicity and temperature stresses that might underlie a prooncogenic progression.

## 1. Introduction

Oral cancer is the sixth-most prevalent human malignancy worldwide, and has a significant 5-year mortality of approximately 50% [1,2,3,4]. The pathogenesis of this cancer includes preventable causes such as tobacco or pan leaf/betel nut chewing and alcohol consumption as well as a spontaneous occurrence in the absence of overt causes [1,2,3,4]. In a large seminal study of approximately 60,000 *asymptomatic* patients presenting at a dental clinic over 12 years, Mashberg and Myers [4] reported in 1976 that, after excluding patients with overt causes (such as tobacco or alcohol), most oral cancer occurred in a U-shaped zone comprising the floor of mouth, especially around the papilla of the submandibular duct, side of tongue, anterior pillar of fauces and retromolar region. They reported that 97% of 207 intraoral malignant lesions detected “prospectively” in such asymptomatic patients occurred in this “high-risk” zone (especially see Figures 3a,b in reference [4]). The authors called for “increased scrutiny” of the underlying cell biology of this region [4]. They, and others, noted that this region comprised the pathway of liquid (and beverage) flow through the mouth, that the lining epithelium in these regions was thin, largely non-keratinized and relatively more permeable than other regions of the mouth [3,4,5,6]. The cellular basis for the anatomic localization of oral cancer occurrence in the high-risk zone in the mouth in the absence of any overt causes, as recognized 50 years ago, remains incompletely understood [1,2,3,4]. Possibilities investigated have included changes in the oral microbiome, including bacterial, fungal and viral agents (including papilloma and Epstein–Barr viruses) ([2,3,7,8,9], and citations therein). Molecular drivers implicated in pathogenesis of oral cancer included the increased activation of prooncogenic p-STAT3, mutations in p53 and Rb, and activity of the E6 protein of human papilloma virus (reviewed in [3]). Moreover, we discovered that cancer-associated mutations in p53 enhanced production of interleukin-6 (IL-6) and activation of p-STAT3 [10,11,12]. Importantly, we discovered that p-STAT3 in cancer cells formed cytoplasmic and nuclear biomolecular condensates which rapidly disassembled in cells exposed to hypotonic conditions, and reassembled when cells were shifted to isotonicity [12,13]. In as much as saliva exiting the submandibular duct is hypotonic (approximately 100 mOsm, i.e., approximately one-third tonicity compared to plasma) ([3,4,5,6], and citations therein), we wondered whether tonicity-driven changes in condensate cell biology in the mouth might drive pathogenesis of oral cancer. The present study provides a new basis for understanding subcellular changes in oral epithelial cells anatomically located along the liquid transit pathway through the mouth in terms of rapid dynamic changes in the cell biology of biomolecular condensates that might drive oncogenicity. Remarkably, we note that while this manuscript was under review, Peng et al. [14] reported that the protein SRSF9 mediated oncogenic splicing of SLC37A4 via liquid–liquid phase separation to promote oral cancer progression.

Indeed, over the last 15 years, phase-separated membraneless biomolecular condensates in the cytoplasm and the nucleus have emerged as providing scaffolding for and modulating diverse subcellular functions [15,16,17,18,19,20,21]. More recently, condensate droplet formation during cellular stress responses, the regulation of translation and transcription, cancer pathogenesis through Tyr-phosphorylated STAT3 condensates and fusion oncoproteins leading to aberrant prooncogenic signaling, involvement of condensates in mechanisms of innate and adaptive immunity, cytokine signaling, viral replication and antiviral mechanisms, and the targeting of condensates by cancer therapeutic agents have been highlighted by numerous investigators [12,13,14,15,16,17,18,19,20,21]. Heterogeneous regions of structure and function within the same condensate (the nucleolus is an eminent example [15,16,17,18]) as well as diversity of function among similar condensates (inhibition vs. stimulation of mRNA translation in condensate granules [22]) add to the significance of biomolecular condensates in modulation of cellular functions. Physiologically, in the intact cell, condensates show dramatic metastability through mechanisms that regulate changes in component concentrations in condensed vs. dispersed phases, as well as changes in condensate size and shape (such as a spheroid to fibril transition) [16,17,23]. Various RNA- and DNA-containing viruses also use phase-separated structures in the cytoplasm or nucleus as locations for viral replication ([17,23,24,25,26,27,28,29,30,31] for detailed citations).

**Figure 1:**
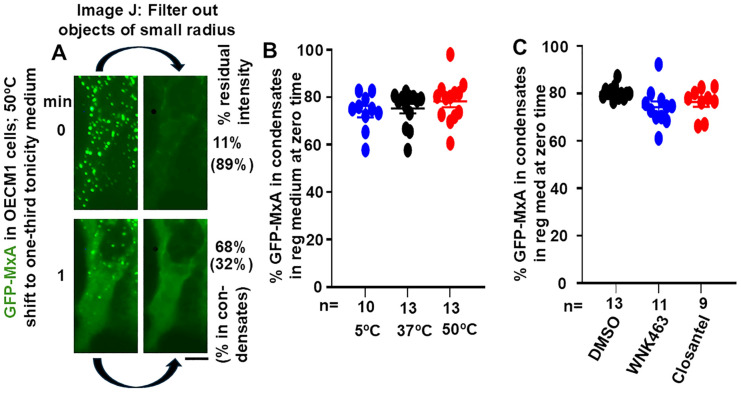
Quantitation of GFP-MxA in condensed vs. dispersed phases. Panel (**A**) illustrates the upper left images from Figure 2A, without and with filtering out small objects of radius < 4 pixels using ImageJ-win64. The GFP intensities over each cell image was quantitated to give % residual intensity, and these data converted to % of GFP-MxA in condensates as indicated in parentheses. Panels (**B**,**C**), multiple cells (n = 9–13) at the start “zero” time in the experiments shown in Figures 3 and 5 were evaluated one hour after the appropriate exposure of cultures in isotonic culture medium. Horizonal lines are Mean ± SE. There was little significant difference between each of the three groups in Panels (**B**,**C**) using ANOVA (Kruskal–Wallis) with Dunn’s post hoc test for multiple comparisons; *p* > 0.05 for all comparisons.

Five years ago, we realized that the cytoplasmic structures formed in human Huh7 hepatoma cell line by the interferon (IFN)-induced antiviral “myxovirus resistance protein” (MxA alias Mx1), a dynamin-family large GTPase (approx. 60 kDa), were membraneless biomolecular condensates of variable size and shape (but, mainly spheroidal) [17,23,32]. Human MxA is an exclusively cytoplasmic protein which has broad-spectrum antiviral activity against various RNA- and DNA-containing viruses [33,34,35,36,37,38,39,40,41,42,43,44,45,46,47]. Contrary to some earlier reports, MxA does not associate with intracellular membranes such as the endoplasmic reticulum (reviewed in [17,23] and citations therein). For our present considerations, MxA inhibited orthomyxoviruses such as influenza A virus and Thogota virus (these have a nuclear step in their life cycle), and the rhabdovirus vesicular stomatitis virus (VSV) (this replicates entirely in the cytoplasm) [33,34,35,36,37,38,39]. In cell-free assays, recombinant MxA inhibited VSV transcription, indicative of the likely targeting of “early” viral transcription by MxA in the infected cell [37,38]. Also, in cell-free assays, recombinant MxA showed the ability to form oligomers, multimers and larger oligomeric structures affected by “salt” concentration in the buffers used (longer fibrils were observed at lower NaCl, 50 mM) [40]. In the cell cytoplasm, MxA formed large granular structures in IFN-treated cells or upon accumulation of exogenously expressed protein [41,42,43,44,45]. Mutants of MxA lacking GTPase activity, and thus lacking antiviral activity, still formed granular structures in the cytoplasm [42,43,44,45]. Critically, it was recognized very early that the L612K mutant of MxA, which remained dispersed in the cytoplasm, retained antiviral activity towards VSV and Thogota virus [46]. Thus, in the case of MxA, the dispersed phase (which can contain GTPase-competent dimers) was viewed as antivirally active, and the granules as a storage reservoir [46,47,48]. Formal quantitation of GFP-MxA in condensed vs. dispersed phases, even in cell images appearing to show “almost all” GFP-MxA in condensates, revealed that 10–25% of the GFP-MxA was typically in the dispersed phase, likely accounting for the antiviral phenotype of such cells (see Figure 1 below) and references [17,48,49]. Clearly, mechanisms that regulate the equilibrium of MxA between condensed and dispersed phases are relevant to understanding its antiviral mechanisms.

We had already observed that cytoplasmic MxA condensates showed rapid disassembly in 1–2 min in Huh7 hepatoma cells exposed to hypotonic medium, and rapid reassembly (also in 1–2 min) in cells shifted back to isotonic medium [23]. Additionally, mechanical pressure and oxidative stress (e.g., by placement of a coverslip on live cells, nitric oxide scavenging or exposure to dynasore) triggered a dramatic transition of GFP-MxA spheroids to motile fibrils in live cells [23]. Thus, a focus of the present studies was to investigate the biochemical mechanisms that underlie the dynamic disassembly and spontaneous reassembly processes and shape changes in diverse condensates in oral cells that taken together might provide insight into pathogenesis of oral cancer, in the absence of overt causes [1,2,3,4]. In as much as the MxA is known to be constitutively present in normal human gingiva [50], at the very least, the antiviral GFP-MxA served as an excellent reporter of condensate dynamics in these processes in oral cells [23,48,49].

For the current investigation, we selected a common circumstance that applies to all of us. We all episodically imbibe cold and warm drinks such as water, tea or coffee everyday, subjecting our oral epithelial cells to stresses of hypotonicity and temperature. MxA is *constitutively* expressed in healthy human gingival epithelium, and is also induced by Type III interferons (IFNs) such as IFN-λ1 in primary gingival cells [48,50]. Moreover, fibrillar MxA structures are observed in the cytoplasm of oral epithelial cells in inflammatory lesions [51]. Typically, only IFN-λ (a Type III IFN) but not IFN-α (a Type I IFN) induce MxA in gingival epithelial cells [52]. Thus, the Type III IFNs comprising the IFN-λ species which induce MxA contribute to antiviral barrier immunity at the level of the oral cavity [48,51]. The realization that oral epithelial cells are constantly bathed in hypotonic saliva (normally one-third tonicity, approximately 100 mOsm, compared to plasma which is 300 mOsm) and are repeatedly exposed to environmental stresses of hypotonicity, temperature and pH (cold and warm drinks of different tonicity = water, tea and coffee are 30–50 mOsm) led us to investigate the biochemical mechanisms that underlie GFP-MxA condensate dynamics in an oral squamous cell cancer line (OECM1) [48,52,53,54,55,56]. These cells in culture grew in tight epithelial sheets, and retained the original property of responding selectively to IFN-λ1 by increasing MxA granules, but not to IFN-α2 [48]. Moreover, unlike primary gingival keratinocytes, OECM1 cells were readily transfected. Exogenous GFP-MxA as well as IFN-λ1 induced endogenous MxA expressed in these cells also retained the property of disassembly of GFP-MxA condensates within 1–2 min of exposure to saliva-like one-third hypotonicity (100 mOsm), and spontaneous reassembly in the next 5–7 min even when continues in saliva-like hypotonicity [48]. Additionally, GFP-MxA condensate containing cells in OECM1 cultures exposed to Lipton’s tea (35 mOsm) or Colombian coffee (50 mOsm) at 37 °C showed rapid condensate disassembly in 1–3 min [48]. That left open the question of the effect of temperature on this process (as in exposure to cold (5 °C) or warm (50 °C) drinks) and the biochemical mechanisms mediating this process as well as recovery from such stress that might function along the fluid intake channels in the mouth.

For the present studies we used an image processing method to separate GFP-MxA in condensate objects vs. dispersed phase in OECM1 cells in order to quantitatively investigate the effect of temperature on hypotonicity-triggered condensate disassembly and spontaneous reassembly process [17,48,49]. The involvement in this process of the WNK-SPAK/OSR1 serine/threonine kinases (WNK, With-No-Lysine kinases; SPAK, STE 20/SPS1-related proline alanine-rich kinase; OSR1, oxidative stress response kinase 1), which are known to regulate aquaporins and ion transporters and co-transporters which mediate water and Na, K and Cl influx and efflux in the kidney [57,58,59,60,61,62], was investigated using pharmacological tools (the pan-WNK inhibitor WNK463, the WNK1-selective inhibitor WNK-IN-11 and the SPAK/OSR1 inhibitor closantel) [61]. Unexpectedly, in these studies, we discovered that the WNK1-selective inhibitor (WNK-IN-11) triggered dramatic and rapid (within 1 h) spheroid to fibril transition of GFP-MxA condensates in live cells. This observation allowed for a test of the antiviral phenotype against VSV of cells with fibrillar GFP-MxA vs. spheroidal GFP-MxA, and more generally, the relationship between different condensates of wild-type GFP-MxA and the antiviral state in single cells.

## 2. Materials and Methods

### 2.1. Cells and Cell Culture

Human oral carcinoma cell line OECM1 was purchased from Millipore-Sigma (St. Louis, MO, USA). Human hepatoma cell line Huh7 was a gift from Dr. Charles M. Rice, The Rockefeller University (New York, NY, USA) [23]. Human lung adenocarcinoma cell line A549 was obtained from the ATCC (Manassus, VA, USA). The respective cell lines were grown in DMEM (Corning Cat. No. 10-013-CV, with glutamine, Na-pyruvate and high glucose) supplemented with 10% *v*/*v* fetal bovine serum (FBS; Gibco, Grand Island, NY, USA) in T25 flasks, 35 mm dishes without or with cover-slip bottoms [23,48,49].

### 2.2. Plasmids and Transient Transfection

The GFP (1-248)-tagged full-length human MxA was a gift from Dr. Jovan Pavlovic (University of Zurich, Zurich, Switzerland) [23,43]. Transient transfections were carried out using the Polyfect reagent (Qiagen, Germantown, MD, USA) and the manufacturer’s protocol [23,48,49].

### 2.3. Live-Cell Fluorescence Imaging

Live-cell imaging of GFP-MxA structures in transiently transfected cells was carried out in cells grown in 35 mm plates using the upright the Zeiss AxioImager 2 equipped with a temperature-regulated stage (range: 5–50 °C) and a 40× water immersion objective with data capture in a manual time-lapse mode (using Axiovision 4.8.1 software) [23,48,49]. Additionally, live-cell imaging was also carried out by placing a coverslip on the sheet of cells and imaging using a 100× oil immersion objective [23,48,49]. Bright-field imaging was carried out using diffraction illumination built into the AxioImager 2 microscope.

### 2.4. Phase Transition Experiments and Fluorescence Imaging

Live GFP-MxA expressing cells in 35 mm plates were imaged using a 40× water immersion objective 1–3 days after transient transfection in growth medium at 37 °C [23,48,49]. After collecting baseline images of MxA condensates at 37 °C, the cultures were exposed to full growth medium for approximately 1 h equilibrated at 5 or 37 or 49–50 °C and imaged (with the stage set at the relevant temperature) (the “0 min” time). Subsequently the cultures were shifted to 1:3 or 1:6 medium (adjusted with sterile water) at the indicated temperatures, followed by time-lapse microscopy of the same filed of cells for the next 15–20 min. For inhibitor experiments, cultures were exposed to the indicated inhibitor in full medium at 37 °C for approximately 50–60 min, imaged, and then shifted to one-fourth tonicity medium at 37 °C containing the same inhibitor followed by time-lapse microscopy of the same field of cells. Fluorescence was imaged as previously reported [23,48,49].

**Figure 2:**
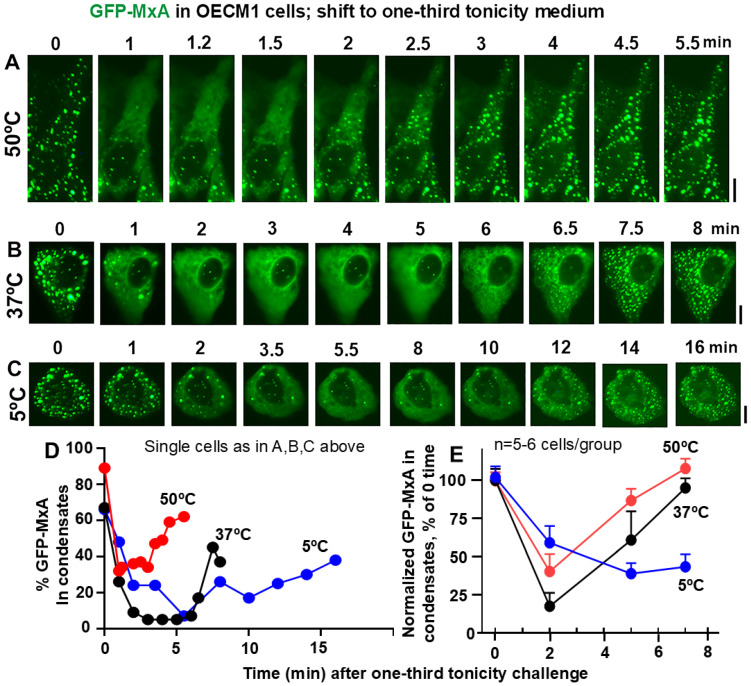
Panels (**A**–**C**), OECM1 cells were either exposed to full culture medium at the indicated temperatures for approx. 60 min, and then shifted to one-third tonicity medium (corresponding to saliva-like hypotonicity; approx. 100 mOsm). Live-cell imaging was carried out as indicated. Scale bar = 10 µm. Panel (**D**), Quantitation of % GFP-MxA per cell in condensates in the same cells shown in Panels (**A**–**C**). Panel (**E**), % GFP-MxA in condensates in 5–6 cells per experimental condition compiled from within the same image frames normalized to that at “zero time” (mean ± SE). The three curves included time-points statistically different from each other (*p* < 0.05) using 2-way ANOVA in Tukey’s post hoc multiple comparisons test.

**Figure 3:**
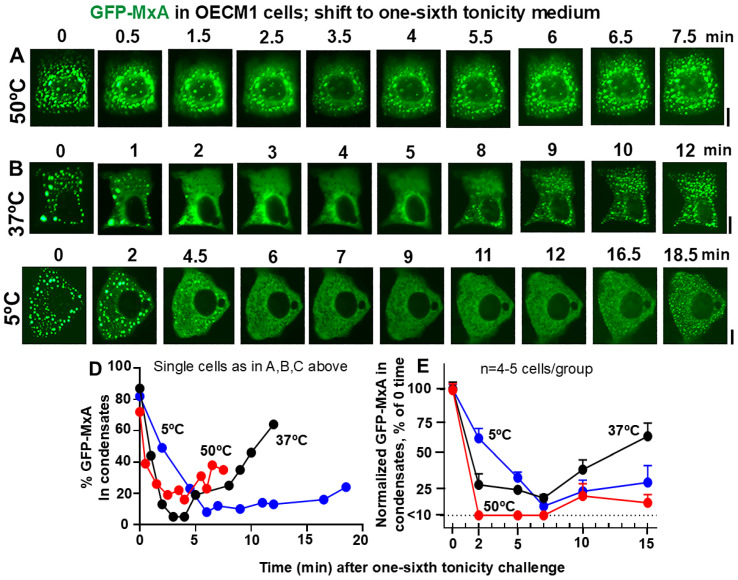
Panels (**A**–**C**), OECM1 cells were exposed to full culture medium at the indicated temperatures for approx. 60 min, and then shifted to one-sixth tonicity medium (corresponding to tea- or coffee-like hypotonicity; approx. 50mOsm). Live-cell imaging was carried out as indicated. Scale bar = 10 µm. Panel (**D**), Quantitation of % GFP-MxA per cell in condensates in the same cells shown in Panels (**A**–**C**). Panel (**E**), % GFP-MxA in condensates in 4–5 cells per experimental condition compiled from within the same image frames normalized to that at “zero time” (mean ± SE). The three curves included time-points statistically different from each other (*p* < 0.05) using 2-way ANOVA in Tukey’s post hoc multiple comparisons test.

**Figure 4:**
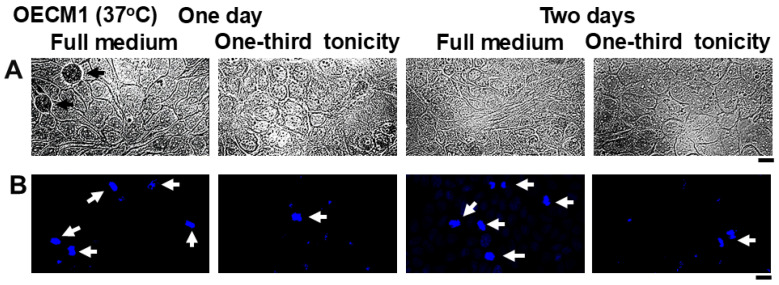
Resistance of OECM1 cell sheets to saliva-like hypotonicity. OECM1 cells were plated in replicate 35 mm dishes (n = 2 per variable) to give nearly confluent sheets the next day. Three days later the fully confluent the cultures were transferred to fresh full medium or to one-third diluted culture medium. One and two days thereafter the live culture sheets in replicate cultures were imaged in bright-field using diffraction illumination (Panel (**A**)) and a water-immersion 40× objective. Thereafter the cultures were fixed and stained using DAPI (Panel (**B**)). Respective images in Panels (**A**,**B**) are from the same culture but different areas. White and black arrows highlight cells in mitosis; from left to right, number of mitoses = 47, 10, 25 and 4 per 10 images under each condition in Panel (**B**). Scale bar = 20 µm.

**Figure 5:**
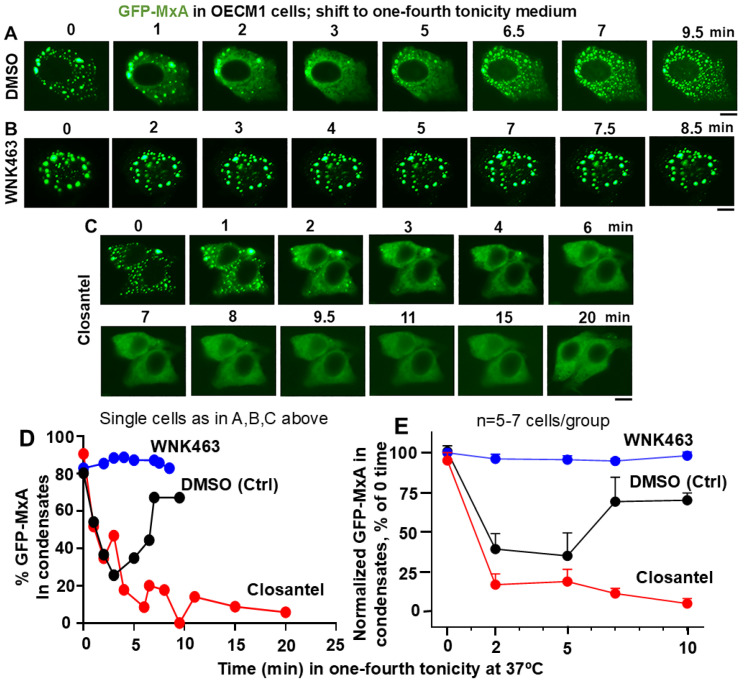
Panels (**A**–**C**), OECM1 cells were either exposed to DMSO, WNK463 (25 µM) or closantel (100 µM) for at least 1 h in full culture medium (approx. 300 mOsm), and then shifted to one-fourth tonicity medium (approx. 75 mOsm) in the continued presence of the inhibitors. Live-cell imaging was carried out as indicated. Scale bar = 10 µm. Panel (**D**), Quantitation of % GFP-MxA per cell in condensates (in the same cells shown in Panels (**A**–**C**)). Panel (**E**), % GFP-MxA in condensates in 5–7 cells per experimental condition compiled from within the same image frames normalized to that at “zero time” (mean ± SE). The three curves included time-points that were statistically different from each other (*p* < 0.05) using 2-way ANOVA in Tukey’s post hoc multiple comparisons test.

Each experimental condition was evaluated in separate 35 mm cultures in at least 3–5 independent experiments. A total of 30–40 individual cells were subjected to time-lapse evaluation for each experimental variable. In Figures 2, 3 and 5, panels A, B and C illustrate representative single cells evaluated under the conditions indicated; Panel D is the specific quantitation derived from those cells illustrated, while Panel E is a summation of 4–7 cells taken from within the same experiment. Survival of cells in sheets in the 35 mm culture plates upon exposure to hypotonic medium was evaluated using bright-field illumination; mitoses in such cultures were evaluated by DAPI staining and counting mitotic figures per 10 images collected using a 40× objective in the AxioImager 2 (Figure 4).

### 2.5. Quantitation of Relative Amounts of GFP-MxA in Condensates vs. Dispersed State in a Cell

Briefly, cell images with mixed condensate and dispersed GFP-MxA were subjected to Filter processing to subtract (“Minimize”) objects of small radii (2–4 pixels) using ImageJ software [17,48,49]. The pixel radius (in the range 2–5 pixels) used for the subtraction was optimized to subtract all condensates from the image. GFP-MxA intensity in the residual subtracted image corresponded to the dispersed protein; subtracting this from the total intensity per cell gave the % of MxA in condensates on a per cell basis [48,49]. When multiple cells were evaluated within the same time-lapse experiment, data from 4 to 7 cells per experimental condition were expressed as % GFP-MxA in condensates normalized to that in condensates per cell at 0 time as 100% (see Figures 2E, 3E and 5E). Each experiment was replicated 3–4 independent times for a total of 20–40 cells evaluated per experimental condition.

### 2.6. VSV Stock and Virus Infection

A stock of the wild-type Orsay strain of VSV (titer: 9 × 10^8^ pfu/mL) was a gift from Dr. Douglas S. Lyles (Dept. of Biochemistry, Wake Forest School of Medicine, Winston-Salem, NC, USA). Single-cycle virus infection studies at high multiplicity (moi > 10 pfu/mL) were carried out essentially as described by Carey et al. [63], as summarized in Davis et al. [23] and Sehgal et al. [64].

### 2.7. Antibody Reagents and Chemicals

Rabbit pAb to human MxA (H-285) (ab-95926) was purchased from Abcam Inc. (Cambridge, MA, USA); mouse mAb to the VSV nucleocapsid (N) designated 10G4 was a gift from Dr. Douglas S. Lyles (Wake Forest School of Medicine, NC, USA). Respective AlexaFluor 488- and AlexaFluor 594-tagged secondary donkey antibodies to rabbit (A-11008 and A-11012) or mouse (A-21202 and A-21203) IgG were from Invitrogen Molecular Probes (Eugene, OR, USA).

The pan-WNK kinase inhibitor WNK463 (which inhibits all four WNK1-4 enzymes) and the SPAK/OSR1 kinase inhibitor closantel were purchased from Sigma-Aldrich (St. Louis, MO, USA), while the WNK1-selective inhibitor WNK-IN-11 was obtained from Cayman Chemical Company (Ann Arbor, MI, USA). These were dissolved in DMSO as 10 mM stocks.

### 2.8. Statistical Testing

The software used was GraphPad Prism (version 7.04 and version 10). Testing of data in Figure 1B,C were carried out using non-parametric ANOVA (Kruskal–Wallis) with Dunn’s post hoc test for multiple comparisons. Testing for the differences in the disassembly and reassembly data points along curves in Figures 2E, 3E and 5E were carried out using the 2-way ANOVA in Tukey’s post hoc multiple comparisons test (*p* < 0.05 was taken as statistically significant).

## 3. Results

### 3.1. Quantitation of GFP-MxA in Condensed vs. Dispersed Phases at the Single-Cell Level

Live-cell fluorescence imaging of transiently expressed wild-type GFP-MxA in OECM1 cells appears to show GFP-MxA almost exclusively in condensates (Figure 1A, upper left panel). However, this is a visual effect not sustained by formal quantitation; there is always GFP-MxA in the dispersed phase (Figure 1A). We carried out formal estimation of GFP-MxA in condensed vs. dispersed phases at the single-cell level using subroutines in ImageJ (Figure 1A, and references [17,48,49]). Green fluorescence images were converted to 16-bit grayscale images, and average intensity was Measured over a cell outline and in a background region to provide total cell intensity. The Filter routine was used to Subtract small objects of (using “Minimum”) radius in the range of 2–5 pixels to delete spheroidal condensates, and the average intensity of the residual filtered image estimated (again, following background subtraction in an area of free plastic away from the cell outline). The residual intensity corresponded to GFP-MxA in the dispersed phase. This can be expressed as % of total intensity in dispersed phase, or its converse as % GFP-MxA in condensates (after subtraction from 100).

Figure 1A illustrates how this was carried out using the first two upper left panels from Figure 2A. Data in Figure 1B show that in normal OECM1 cells kept at 37 °C in isotonic culture medium, approximately 20–25% of GFP-MxA in cells was located in the dispersed phase (range 10–40%). Thus, even in cells whose images gave the visual appearance of an almost exclusive localization of GFP-MxA in condensates, significant GFP-MxA was present in the dispersed phase. The data in Figure 1B also show that the partitioning of GFP-MxA between condensed vs. dispersed phases was largely unaffected between cultures kept for approximately 1 h in isotonic medium at 5, 37 or 50 °C. Similarly, Figure 1C shows that this partitioning was largely unaffected in cultures kept for approximately 1 h at 37 °C in isotonic medium containing the WNK-SPAK/OSR1 pathway inhibitorsFor WNK463 (25 µM) or closantel (100 µM), the images tabulated in Figure 1B,C correspond to “zero-time” partition estimates of single cells (n = 9–13) from the experiments in Figures 2, 3 and 5 just before these cultures were challenged with hypotonic medium.

### 3.2. Temperature Sensitivity of the Spontaneous Reassembly of GFP-MxA Condensates Dispersed by a Hypotonic Challenge

For economy of illustration, Panels A, B, C and D in Figures 2, 3 and 5 show detailed images and quantitation of the initial image and the subsequent full time-lapse sequence of the same representative single cell for each of the experimental variables investigated. Each of these experiments were replicated 3–4 times and time-lapse data were collected for 20–40 cells per variable. From within this set, Panels E in Figures 2, 3 and 5 illustrate the sum of quantitation (mean ± SE) derived from 4 to 7 cells per indicated experimental condition which were located in the same image frames. Statistical evaluation was carried out using 2-way ANOVA using Tukey’s multiple comparisons. Importantly, all imaging data with respect to each of the variables illustrated within Figures 2, 3 and 5 were collected within 2–3 h on the same day using replicate cultures plated and transfected at the same time as part of the same experiment.

The rationale for the experiment in Figure 2 was to investigate GFP-MxA condensate dynamics in oral cancer cells in cultures shifted from isotonic medium (approximately 300 mOsm) to saliva-like hypotonicity (“one-third tonicity” corresponding to approximately 100 mOsm). In Figure 3 the rationale was to shift oral cells from isotonic medium (300 mOsm) to beverage-like hypotonicity (“one-sixth tonicity” corresponding to approximately 50 mOsm). In each experiment, temperatures corresponding to cold (5 °C) or warm (49–50 °C) drinks were compared to 37 °C.

Data in Figure 2B,D,E recapitulate our previous observation [48] that exposure of oral epithelial cells to one-third hypotonicity at 37 °C caused a rapid disassembly of GFP-MxA condensates within 1–2 min followed by spontaneous reassembly 6–7 min later. This process was sped up at 50 °C—rapid disassembly in 1–2 min with reassembly in 2–3 min (Figure 2A,D,E). At 5 °C, both disassembly and reassembly were slowed and remained significantly incomplete for >10 min (Figure 2C–E). Thus, overall, both disassembly and reassembly were temperature sensitive.

Challenging GFP-MxA condensate-containing cells with beverage-like one-sixth hypotonicity also showed a temperature sensitivity of the disassembly/reassembly processes (Figure 3). At 37 °C, the rapid disassembly was followed by reassembly 9–12 min (Figure 3B,D,E). In comparison, disassembly was faster at 50 °C (by 2 min) while reassembly could be more variable (compare the 50 °C lines in Figure 3D,E); it remained incomplete in particular cells even at 15 min (Figure 3E). However, at 5 °C, both disassembly and reassembly were slowed in all cells (Figure 3D,E). It is noteworthy that the temperatures tested did not change the partitioning of GFP-MxA into condensed vs. dispersed phases in isotonic culture medium for >1 h (Figure 1B), but that the active process of reassembly during the regulated volume decrease (RVD) part of the cell response to hypotonic challenge was particularly temperature sensitive at 50 °C and 5 °C.

### 3.3. Resistance of Oral Carcinoma Cells to Saliva-like Hypotonicity

In carrying out the above experiments, we observed that cultures comprising confluent sheets of oral squamous carcinoma cells (OECM1) were remarkably resistant to one-third tonicity medium for at least up to one day without disruption of the cell sheet (Figure 4A, left panels), but with some decrease in the level of mitosis (Figure 4B, left panels). By two days we still observed a largely intact cell sheet but with decreased levels of mitosis (Figure 4A,B, right panels). These observations suggested that OECM1 cells likely possessed biochemical mechanisms to protect from and survive sustained hypotonicity stresses such as in the mouth. We have followed the lead of renal physiologists by asking whether the same biochemical mechanisms (in particular the WNK-SPAK/OSR1 serine-threonine kinase pathway) which regulates water, K, Na and Cl fluxes into and out of renal tubular cells (57–62) might also function in oral cells.

### 3.4. Involvement of the WNK-SPAK/OSR1 Kinase Pathway in the Dynamic Response of GFP-MxA Condensates to Hypotonicity

Briefly, exposure of cells (such as renal tubular cells) to hypotonicity is known to activate the chloride-sensitive WNK serine-threonine kinases (WNK1-4) (reduced chloride activates the kinase) which phophorylate (and thus activate) upstream aquaporin channels as well as downstream SPAK/OSR1 kinases, which, in turn, regulate Na, K, and Cl cotransporter NKCC1 (which mediates the influx of these ions) and additional targets [59,60,61,62]. Thus, we investigated the effect of the pan-WNK kinase inhibitor WNK463, the WNK1 kinase-selective inhibitor WNK-IN-11 and the SPAK/OSR1 kinase inhibitor closantel on the disassembly and reassembly of GFP-MxA condensates in oral cells challenged with one-fourth strength tonicity (75 mOsm) medium. In these experiments, GFP-MxA expressing OECM1 cultures were first exposed to the relevant inhibitor for approximately 60–110 min at 37 °C, and imaged (as in Figure 1C). These were then challenged with one- fourth tonicity medium at 37 °C containing the same inhibitor followed by time-lapse microscopy.

The data in Figure 5A,D,E confirm the disassembly of GFP-MxA in 2–3 min in cells in control culture exposed to DMSO followed by reassembly beginning in 6–7 min. In comparison, the inclusion of WNK463 inhibited/reduced the disassembly; thus, there was little scope to observe reassembly in these cells (Figure 5B,D,E). In contrast, closantel allowed rapid disassembly but markedly slowed down reassembly (Figure 5C–E). These data implicate the WNK-SPAK/OSR1 kinase pathway in the dynamic regulation of GFP-MxA condensates during hypotonic challenge and recovery from such a tonicity stress. Thus, the cellular biochemistry which regulates water and salt fluxes in the renal tubular cells, appears to be also operative in oral cells (Figure 5) with the additional feature of temperature sensitivity (Figure 2 and Figure 3).

### 3.5. Dramatic and Rapid Spheroid to Fibril Transition of GFP-MxA Condensates in Live Cells Triggered by the WNK1-Kinase-Selective Inhibitor WNK-IN-11

**Figure 6:**
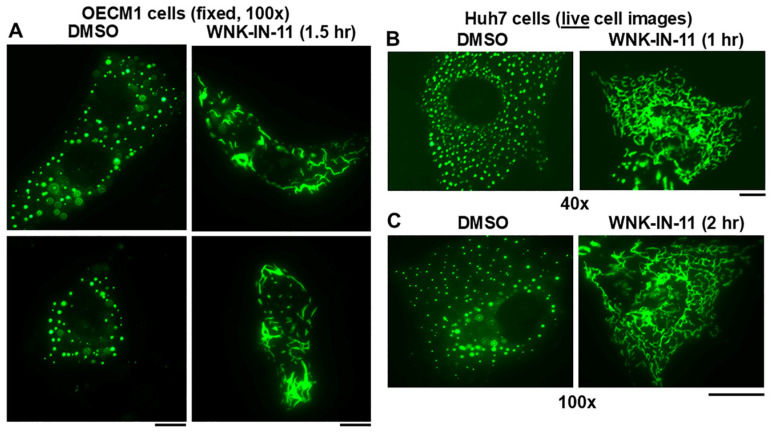
Spheroid to fibril transition of GFP-MxA condensates triggered by WNK-IN-11. Panels (**A–C)**, respectively show representative OECM1 and Huh7 cells expressing GFP-MxA two days after transfection in 35 mm plates and exposed to either DMSO or WNK-IN-11 (100 µM) in isotonic regular medium at 37 °C for the indicated times. Fixed and live cells were imaged using 40× or 100× objectives as indicated. Scale bars = 10 µm.

**Figure 7:**
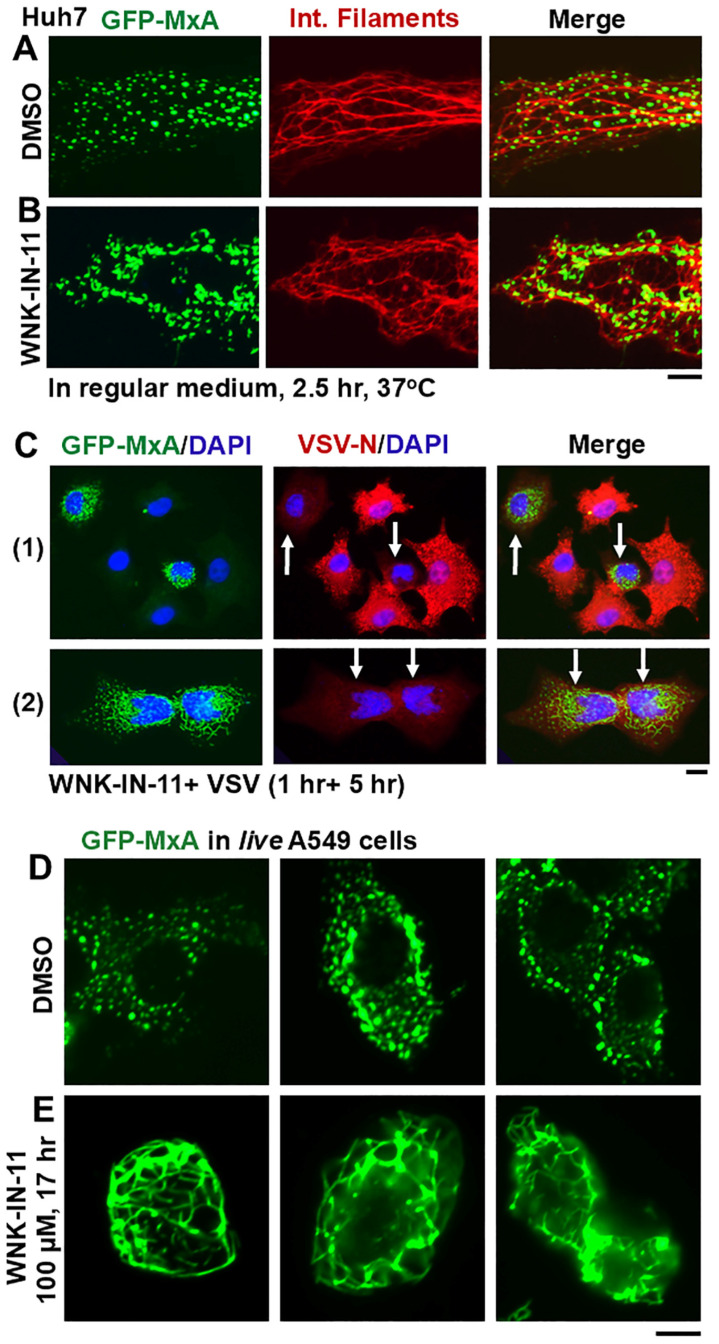
Properties of fibrillar GFP-MxA. Panels (**A**,**B**), Huh7 cells expressing GFP-MxA were treated with DMSO or with WNK-IN-11 (100 µM) for 2.5 h, fixed and then imaged for intermediate filaments. Panel (**C**), a culture of Huh7 cells expressing GFP-MxA was treated with WNK-IN-11 (100 µM) for 1 h and then exposed to VSV (moi > 10 pfu/cell) for 5 h in the continuous presence of the inhibitor. The culture was fixed and imaged for VSV-N protein (red) in cells expressing and not expressing GFP-MxA. Sub panels (1) (showing spheroidal MxA) and (2) (showing fibrillar MxA) are from the same culture. White arrows, cells showing an antiviral phenotype. Panels (**D**,**E**), live A549 cells expressing GFP-MxA and exposed to DMSO or WNK-IN-11 (100 µM) for 17 h were imaged as indicated. Scale bars = 100 µm.

**Figure 8:**
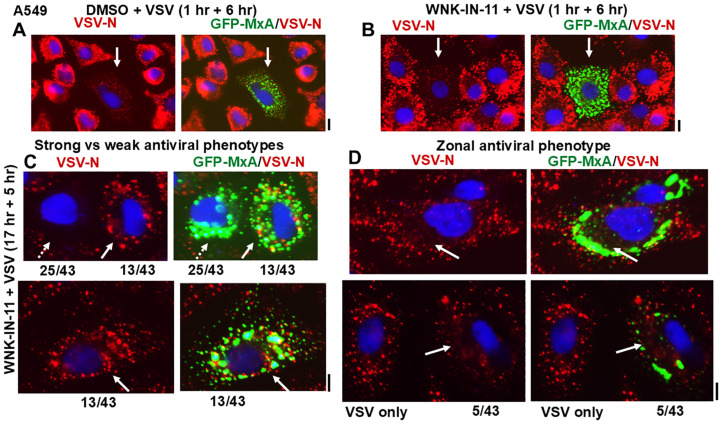
Single-cell antiviral phenotypes of A549 cells containing GFP-MxA structures without or after exposure to WNK-IN-11 for 1 (Panels **A**,**B**) or 17 h (Panel **C**,**D**) and challenged with VSV (moi > 10 pfu/cell) for 6 h (Panels (**A**,**B**)) or 5 h Panel (**C**). In Panels (**A**,**B**), solid white arrows highlight GFP-MxA expressing cells with a strong antiviral phenotype. Panels (**C**,**D**) illustrative images of cells with different antiviral phenotypes out of a set of 43 GFP-positive cells scored. In Panel (**C**), the broken arrow highlights a cell with a strong antiviral phenotype, solid white arrow highlights an adjacent and another cell with little antiviral activity. Panel (**D**) illustrates two cells with irregular GFP-MxA structures showing absence of virus replication in zones of the cell cytoplasm highlighted by longer solid white arrows. For comparison, Panel (**D**) (lower panels) also show a fully infected cell with no GFP-MxA. Scale bars = 10 µm.

Investigation of the WNK1-selective inhibitor WNK-IN-11 (100 µM) [51] in experiments modeled on those in Figure 5 provided an unexpected observation. Live-cell imaging even before carrying out the hypotonic challenge revealed that exposure of GFP-MxA-expressing OECM1 cultures to WNK-IN-11 showed a transition of spheroidal condensates to fibrils. Parenthetically, WNK-IN-11 had a modest effect on one-fourth tonicity triggered disassembly and slowed reassembly). However, the more dramatic effect was a transition of GFP-MxA condensates to a fibrillar phenotype (Figure 6A). This fibrillar phenotype also developed in WNK-IN-11-treated OECM1 cultures kept continuously in isotonic medium, and also in other cancer cell lines (Figure 6B,C and Figure 7C). The data in Figure 6B,C recapitulate the spheroid to fibril transition observed by us previously in GFP-MxA condensates in hepatoma Huh7 cells subjected to oxidative stress (as in Figure 8 in reference [23]). Since we previously reported the association of GFP-MxA condensates with intermediate filaments in Huh7 cells [17,23], we investigated the association of GFP-MxA fibrillar condensates in Huh7 cells with intermediate filaments. As before [17,23], the data in Figure 7A,B show that both spheroidal and fibrillar GFP-MxA condensates in Huh7 cells were located alongside of but distinct from giantin-positive intermediate filaments. The structural transformation of GFP-MxA condensates can be rather dramatic and extensive in live A549 live lung cancer cells exposed to WNK-IN-11 for 17 h (Figure 7E).

### 3.6. Relationship(s) Between GFP-MxA Condensates and the Antiviral Phenotype Against VSV at the Single-Cell Level

The question of whether spheroid to fibril transition affected the antiviral activity of GFP-MxA was addressed in Huh7 cells exposed to WNK-IN-11 (100 µM) for 1 h. While most GFP-MxA expressing cells in such cultures showed a fibrillar transition, significant numbers of cells with spheroidal condensates persisted, allowing for a side-by-side comparison of the antiviral phenotype of cells of both kinds of condensates in the same culture. Such a culture was challenged with VSV at high multiplicity of infection (>10 moi/cell) and then fixed at 5–6 h after beginning of infection followed by immunofluorescence evaluation of the accumulation of the viral nucleocapsid protein (N) [23,24]. Parenthetically, the N protein accumulates in the cytoplasm in phase-separated condensates which form centers for viral replication [24]. Figure 7(C1,C2) shows images from the same culture verifying, first, that cells with spheroidal GFP-MxA condensates showed a clear antiviral phenotype (white arrows subpanel (1)) and, second, that cells with fibrillar GFP-MxA also showed a strong antiviral phenotype (white arrows in subpanel (2)).

These studies were extended to A549 cells which had shown the development of large heterogeneous GFP-MxA structures following exposure to WNK-IN-11 for 17 h (Figure 7E). The data in Figure 8A,B summarize that when A549 cells were treated with WNK-IN-11 for only 1 h prior to a VSV challenge all GFP-MxA condensate-containing cells showed a strong antiviral phenotype irrespective of the structure of the condensates. However, when cells were exposed to WNK-IN-11 overnight (17 h) and then challenged with VSV, anomalous antiviral phenotypes became apparent. While the majority of GFP-MxA-containing cells (25/43 in this culture) showed a strong antiviral phenotype (Figure 8C shows one example; broken arrow), a subset (13/43) showed the simultaneous presence of GFP-MxA condensates together with VSV-N condensates intermixed throughout the cytoplasm (Figure 8C, white arrows). A smaller subset of cells (5/43) showed zonal areas of the cytoplasm devoid of VSV-N in the vicinity of GFP-MxA structures, with the same cells exhibiting VSV-N structures elsewhere in the cytoplasm (Figure 8D, white arrows). Thus, in these instances, the unit of antiviral phenotype was not the entire cell, but a portion of the cell, perhaps due to zonal restrictions imposed by cytoplasmic viscosity (Figure 8D). The mechanisms which regulate partitioning of GFP-MxA into the dispersed phase may regulate the apparent antiviral activity.

## 4. Discussion

This study represents a melding of principles of human gross anatomy of the mouth with the new cell biology of biomolecular condensates to provide potential novel insights into mechanisms of the pathogenesis of oral cancer. We highlight the observation reported 50 years ago that the sites of occurrence of oral cancer (in patients without other overt causes such as tobacco or alcohol use) follow a U-shaped distribution along the floor of the mouth, sides of the tongue, the anterior fauces and the retromolar region [4]. This represents the liquid transit channel through the mouth and here, the oral mucosa is the thinnest and is the most permeable [1,2,3,4,5,6]. We suggest that the constantly hypotonic saliva together with the repetitive disassembly and reassembly of diverse cytoplasmic and nuclear condensates of prooncogenic transcription factors such as p-STAT3 and fusion oncoproteins in cells along this liquid transit path due to stresses of hypotonicity and temperature contribute to the process of cancer pathogenesis in these locations. In the present study, we used the GFP-tagged human antiviral protein MxA essentially as a reporter for the complex dynamics of condensate assembly in oral cancer cells subjected to environmental stresses of hypotonicity and temperature.

**Figure 9:**
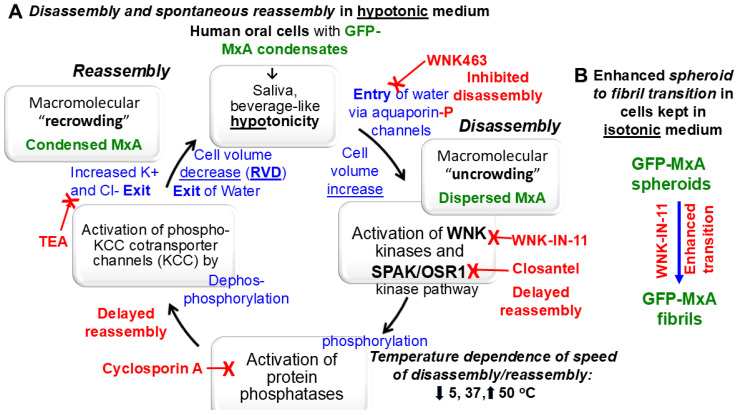
Schematic overview of the WNK-SPAK/OSR1 pathway involved in the dynamic disassembly and temperature-sensitive reassembly of GFP-MxA biomolecular condensates by hypotonicity (Section **A**), and in the spheroid to fibril transition (Section **B**) in oral cancer cells (adapted from Figure 9 in reference [48]). KCC, potassium-chloride cotransporter channels 1–4; OSR1, oxidative stress regulated kinase 1; SPAK, Ste-family proline and alanine enriched kinase; TEA, tetraethylammonium chloride; RVD, regulated volume decrease; WNK kinase, “With no lysine” kinase family members 1–4.

In previous experiments, we had observed that cytoplasmic GFP-MxA condensates in oral epithelial cells were exquisitely sensitive to saliva- and beverage-like hypotonicity (range 30–100 mOsm)—exposing oral epithelial cells to hypotonicity rapidly disassembled the condensates within 1–3 min. This then triggered an active cellular process which mediated a “spontaneous” reassembly of MxA into a different set of condensates in the next 5–10 min. This process reflected water influx and “uncrowding” of the cytoplasm in the disassembly phase, and water efflux and “recrowding” of the cytoplasm in the reassembly process—a regulated volume decrease (RVD) (Figure 9) [48]. The earlier observation by us that the protein phosphatase inhibitor cyclosporin A (which inhibits PTP2B alias calcineurin) and tetraethylammonium chloride (TEA) (which inhibits K and Cl efflux through KCC co-transporters, and thus water efflux) inhibitor markedly slowed down GFP-MxA reassembly into condensates [48] suggested the involvement of the chloride-sensitive WNK kinase-SPAK/OSR1 kinase-PTP-KCC pathway in condensate cycling in oral cells (Figure 9). It was previously unknown to what extent this pathway was sensitive to temperature since the prior renal physiologists always worked in cells at the internal body temperature of 37 °C [57,58,59,60,61,62].

In the present experiments, we observed that in addition to tonicity, the cycling of GFP-MxA condensates through disassembly and reassembly following a saliva- or beverage-like hypotonicity challenge was indeed temperature sensitive—faster at 50 °C (corresponding to a warm drink) and slower at 5 °C (corresponding to a cold drink) (Figure 2 and Figure 3). It was mainly the recovery from a hypotonic challenge that was particularly temperature dependent, not so much the partitioning in regular isotonic medium at different temperatures (Figure 1).

Water and chloride are known to be allosteric inhibitors in osmosensing by WNK kinases [57,58,59,60,61,62]. For the present study, we surmised that hypotonicity, which causes water influx through aquaporin channels, would lower cytosolic chloride concentration leading to activation of the WNK kinases and thus activation of downstream targets, eventually leading to activation of the downstream KCC2 co-transporter and consequent efflux of water and thus volume restoration (Figure 9). This possibility was investigated in hypotonicity-driven regulation of GFP-MxA condensate disassembly and reassembly using well-characterized small-molecule inhibitors of the WNK-SPAK/OSR1 serine-threonine kinases: the pan-WNK inhibitor WNK463 which inhibits all four WNK1-4 kinases, WNK-IN-11 which is WNK1 selective and closantel which inhibits the SPAK/OSR1 kinases [61]. As background information, phosphorylation of aquaporin channel proteins by WNK kinases contributes to their water-flux activity (Figure 9) [61]. Exposing OECM1 cells to the pan-WNK kinase inhibitor WNK463 for 1 h reduced the ability of hypotonicity to disassemble GFP-MxA, consistent with a reduction in water influx through aquaporin channels (Figure 5B,D,E). In contrast, closantel, an inhibitor of the downstream SPAK/OSR1 serine-threonine kinases did not affect disassembly but markedly slowed down the reassembly process (Figure 5C–E). These data, as in the kidney, implicate the WNK-SPAK/OSR1 pathway in the regulation of biomolecular condensates in the mouth.

WNK1 is known to phosphorylate and thus maintain or enhance the activity of the NKCC1 cotransporter which mediates influx of Na, K and Cl into cells [65,66]. The WNK1-selective inhibitor WNK-IN-11, had only modest effects on the disassembly–reassembly process (it inhibits only one of the four WNK kinases) but caused a dramatic and rapid (within 1 h) spheroid to fibril transition of GFP-MxA condensates in three different cancer cell lines (Figure 6A,B, Figure 7D and Figure 9B). This spheroid to fibril transition was triggered even in cells kept in isotonic medium (Figure 6 and Figure 9B). The new observations showing WNK-IN-11-triggered GFP-MxA fibril formation in Huh7 cells (Figure 6B,C) are similar to our previous findings of the formation of fibrillar GFP-MxA networks in live Huh7 cells subjected to oxidative stress [23]. Additionally, and in confirmation of previous observations in Huh7 cells [23], spheroidal and fibrillar GFP-MxA condensates were located alongside but were distinct from giantin-containing intermediate filaments (Figure 7A,B). The overnight (17 h) exposure of A549 cells produced accumulation of GFP-MxA in larger, even vacuolar, condensates (Figure 7D).

Of relevance here is that Kochs et al. reported in 2002 [40] that in cell-free studies, recombinant MxA self-assembled into long fibrils under “low salt” conditions. In their cell-free assays, “low” NaCl referred to 50 mM [40]. For comparison, in live cell cytosol Na, concentration is at approximately 10–15 mEq [67]; it is possible that the known inhibition of NKCC1 cotransporter activity by WNK-IN-11 [61,68] may “lower” cytosolic Na, triggering a fibrillar transition of GFP-MxA condensates comparable to that reported in Figure 1 in reference [40]. More recently, the data of Imanulli et al. showed that human MxA in the cytoplasm of oral epithelial cells in mucosal lesions of graft versus host reaction (GVHD) were fibrillar in structure [50].

Biologically, to a first approximation, cells with fibrillar or larger mesoscale GFP-MxA structures of any shape retained a strong antiviral phenotype towards VSV (Figure 7C and Figure 8A,B). However, the anomalous antiviral phenotypes illustrated in Figure 8C suggest involvement of cellular functions in regulating the antiviral activity of GFP-MxA—not all WNK-IN-11-treated A549 cells containing GFP-MxA condensates exhibited an antiviral phenotype (Figure 7C). The underlying biochemistry is likely to involve as yet unknown effects of this kinase inhibitor on the partitioning of GFP-MxA between the condensed (storage) and dispersed (antivirally active) phases.

WNK kinases and the downstream SPAK/OSR1 kinases are implicated in the migration, proliferation and metastasis of human gliomas, hepatocellular carcinoma, breast cancer and colon cancer [61,68,69,70]. In the activation of the WNK pathway in gliomas, there is a direct relationship between cell volume preservation by the WNK-SPAK/OSR1 pathway components and tumor aggressiveness [68,70]. However, none of the studies thus far have considered alterations of relevant signaling through changes in biomolecular condensates mediated by aberrant activation of the WNK-SPAK/OSR1 cascade in cancer cells.

A limitation of the present studies is that the human OECM1 oral squamous cancer cell line was used as a representative of the oral epithelium. Although OECM1 cells retain key properties of the oral epithelium (grow in tight sheets, and MxA is induced in these cells by IFN-λ1, a Type III interferon, but not by IFN-α2, a Type I interferon [48]), a next step would be to include additional oral cancer cell lines and to also evaluate primary human gingival or immortalized gingival cell lines in these investigations.

In addition to stresses of tonicity and temperature, the buccal mucosa is also subjected to challenges of pH and to various chemicals in our food and drink. The influence of these on the cell biology of biomolecular condensates in oral cells deserves attention and remains a topic for future investigations.

## 5. Conclusions

All of us repeatedly challenge our oral mucosa with the stresses of tonicity and temperature every single day. These stresses trigger repetitive cycles of disassembly/reassembly of biomolecular condensates in oral epithelial cells. We showed that such cells possessed a biochemical defense mechanism comprising the WNK-SPAK/OSR1 kinase pathway triggered by hypotonicity which ensured rapid cell volume recovery. In studies in live cells, this pathway mediated rapid and dynamic changes in the partitioning of the antiviral human GFP-MxA protein between condensed storage granules and the dispersed antiviral state. Both the disassembly and the reassembly phases of these GFP-MxA biomolecular condensates were sensitive to cold (5 °C) and warm (50 °C) temperatures (slowed down or speeded up, respectively). The data raise the possibility that cold and warm hypotonic drinks (water, tea or coffee) might broadly alter the cytoplasmic and nuclear condensate landscape in oral epithelial cells. Importantly, the data suggest a novel subcellular mechanism-dysfunction of prooncogenic biomolecular condensate biology- to consider in understanding the U-shaped “high risk” zone of cancer occurrence in the floor of the mouth [4]. The data also reinforce that oral hygiene should include gargling with warm salt water for the fastest recovery of the condensate landscape in the oral mucosa.

## Data Availability

All data are available within the manuscript.
